# Resveratrol Downregulated PRDX4 Expression to Inhibit the Progression of Renal Cell Carcinoma via Wnt/β‐Catenin Pathway

**DOI:** 10.1002/fsn3.70352

**Published:** 2025-06-01

**Authors:** Hongzhi Li, Zhun Wang, Xueji Chen, Shuai Li, Fang Zhang

**Affiliations:** ^1^ The First Department of Urological Surgery Cangzhou Central Hospital Cangzhou People's Republic of China; ^2^ Department of Oncology North China Petroleum General Hospital Cangzhou Hebei People's Republic of China; ^3^ Neonatology Department Cangzhou Women and Children's Hospital Cangzhou Hebei People's Republic of China

**Keywords:** PRDX4, renal cell carcinoma, Wnt/β‐catenin pathway

## Abstract

Renal cell carcinoma (RCC) is a common urological cancer. Resveratrol, a natural plant polyphenol, has demonstrated antitumor efficacy in various human cancers. Despite its recognized potential, the specific impact of resveratrol on RCC remains unclear. This study aimed to ascertain the potential of resveratrol in suppressing RCC progression and to elucidate the intricate molecular mechanisms governing its effects. The CCK8 assay outcomes indicate that resveratrol inhibits RCC cell viability in a dose‐dependent manner. Transwell and wound healing assays confirmed the inhibitory effects of resveratrol on RCC cell invasion and migration. Flow cytometry further demonstrated that resveratrol induced apoptosis in RCC cells. Additionally, in vivo experiments have established the ability of resveratrol to impede RCC tumor growth. Mechanistically, resveratrol exhibits its inhibitory influence on RCC cells by downregulating PRDX4 expression, consequently weakening the Wnt/β‐catenin pathway. These findings gain support from experiments utilizing a Wnt/β‐catenin pathway activator. In summary, our results suggest that resveratrol impedes the progression of RCC by inhibiting the Wnt/β‐catenin signaling pathway via the downregulation of PRDX4.PRDX4.

## Graphical abstract

This study demonstrates that resveratrol inhibits the activation of the Wnt/β‐catenin signaling pathway by downregulating PRDX4 expression, thereby suppressing renal cell carcinoma (RCC) progression. In vitro and in vivo experiments confirmed that resveratrol reduces RCC cell viability, migration, invasion, and tumor growth while promoting apoptosis. Mechanistically, PRDX4 silencing enhances WIF1‐mediated inhibition of Wnt/β‐catenin signaling, revealing PRDX4 as a potential therapeutic target and biomarker for RCC.

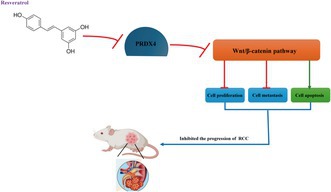

## 1 Introduction

Renal cell carcinoma (RCC) is a prevalent and lethal malignancy within the urinary system, contributing to approximately 140,000 fatalities globally on an annual basis (Ljungberg et al. 2022; Peng et al. 2020). Although the precise cause of RCC remains elusive, various factors, such as heredity, smoking, and hypertension, have been implicated in its pathogenesis (Guo and Wang 2016). Notably, RCC is resistant to conventional treatments, including radiotherapy, chemotherapy, and biological targeted therapy, making surgery the sole curative option (Chen et al. 2022; Wang et al. 2023). Unfortunately, the prognosis of patients with RCC remains poor because of the limited adoption of screening protocols and the concealment of clinical manifestations during the early stages of the disease (Yu et al. 2020). Presently, the survival rate for patients with RCC falls below 10% over a 5‐year period (Fu et al. 2017). Consequently, there exists an urgent imperative to develop more efficacious drugs for RCC treatment.

In recent years, there has been a growing interest in exploring the use of naturally occurring compounds to inhibit cancer development (Juan et al. 2002). Resveratrol, a natural polyphenol compound abundantly found in various sources like peanuts, grapes, and mulberries, has garnered attention for its promising medicinal properties (Tian et al. 2020). Through the regulation of cancer‐related gene expression, resveratrol has demonstrated anticancer potential across diverse human cancer types, including bladder cancer, liver cancer, prostate cancer, and ovarian cancer (Rauf et al. 2018; Jiang et al. 2017; Bai et al. 2017). The observed anticarcinogenic effects of resveratrol are believed to be linked to its antioxidant capacity (Zheng et al. 2018). Jiang et al. demonstrated that resveratrol could reduce the levels of reactive oxygen species (ROS) through the ERK/MAPK signaling pathway, thereby impeding the development of human diffuse large B‐cell lymphoma (Li et al. 2017). Recent studies have suggested that resveratrol inhibits RCC progression via multiple pathways. Resveratrol induces S‐phase arrest in a dose‐dependent manner, significantly increasing the S‐phase cell population and reducing the G0/G1‐phase cell population, thereby restricting proliferation (Taheri et al. 2024). Resveratrol also suppresses epithelial‐to‐mesenchymal transition in RCC by inhibiting HIF‐1α transcriptional activation, which reduces cellular migration and invasion (Kim et al. 2016). Additionally, it inhibits the activation of STAT3 and STAT5 pathways, enhances sorafenib‐induced apoptosis in RCC cells, and suppresses the malignant progression of RCC (Wang et al. 2024). However, the precise molecular mechanisms underlying the inhibitory effects of resveratrol on RCC remain unclear.

PRDX4 is a crucial antioxidant enzyme with ubiquitous expression in mammalian cells (Rhee et al. 2005) and plays a pivotal role in mitigating oxidative stress primarily by neutralizing H_2_O_2_ (Zheng et al. 2020). Given the established link between oxidative stress and the advancement of cancer, there has been a thorough investigation into the involvement of PRDX4 in tumors (Reuter et al. 2010). PRDX4 upregulation has been reported in various cancers, including lung and ovarian cancers, glioblastoma, and hepatoblastoma (Mizutani et al. 2019; Pylväs et al. 2010; Kim et al. 2012). Notably, a recent study focusing on RCC revealed a significant correlation between elevated levels of PRDX4 and a poor prognosis for RCC patients (Kocatürk 2023). Considering the robust antioxidant capabilities of resveratrol, we hypothesized that it impedes RCC development by downregulating PRDX4 expression.

In this study, we initially demonstrated the therapeutic potential of resveratrol in RCC by confirming its inhibitory effects on malignant phenotypes of RCC cells and tumor growth. Rescue experiments subsequently validated the essential role of PRDX4 in resveratrol‐induced inhibition of RCC progression. Finally, we investigated the underlying mechanism by which resveratrol downregulates PRDX4 to impede RCC progression.

## 2 Materials and Methods

### 2.1 Main Reagent

Resveratrol (> 99% pure, Sigma, USA), RPMI‐1640 medium (Gibco, USA), Fetal Bovine Serum (FBS, Hyclone, USA), Vascular Cell Basal medium and Vascular Endothelial Cell Growth Kit‐VEGF (ATCC, USA) Dimethylsulfoxide (DMSO, Sigma, USA), Lipofectamine 2000 (Invitrogen, USA), puromycin (Santa Cruz Biotechnology, USA), CCK8 kit (Beyotime Biotechnology, China), 2,3,5,4′‐Tetrahydroxystilbene‐2‐O‐β‐d‐glucoside (TSG, Sigma, USA), diluted Matrigel (BD Biosciences, USA), Annexin V‐FITC Apoptosis Detection kit (BD Pharmingen), DAB color developing solution (Solarbio, China), hematoxylin (Solarbio, China), Lithium chloride (LiCl, Sigma, USA) NP‐40 IP buffer (Sigma, USA), protein A/G beads (Bimake, USA), TRIzol Reagent (Invitrogen, USA), First‐Strand cDNA kit and SYBR Green kit (Tiangen, China), RIPA and PMSF (Beyotime Biotechnology, China), and BCA kit (Solarbio, China).

### 2.2 Human Tissue Samples

This study was conducted ethically, and approval was obtained from the Ethics Committee of Cangzhou Central Hospital (No. 2022‐115‐02 (z)). Prior to inclusion, explicit informed consent was obtained from all the participants. Human tissues used in this study were sourced from individuals diagnosed with RCC and their corresponding adjacent noncancerous tissues. All clinical tissue samples were promptly stored in liquid nitrogen for subsequent experiments.

### 2.3 Cell Culture

Human RCC cell lines (A498 and Caki‐1) and a normal cell line (HK‐2) were cultured in RPMI‐1640 medium (plus 10% FBS). To maintain sterility, 80 U/mL Penicillin–Streptomycin was added. All cell lines were cultured at 37°C in 5% CO_2_. The medium was replaced every 2 days, and the cells were passaged upon reaching 80% confluence. Resveratrol was dissolved in DMSO at a stock concentration of 100 mM. Subsequently, it was diluted in the culture medium to achieve the desired concentrations (25, 50, 75, and 100 μM). DMSO was used as a control in all experiments.

### 2.4 Cell Transfection

The PRDX4‐overexpression plasmid was constructed using the PcDNA3.1 vector, and small hairpin RNA (shRNA) was used to knockdown PRDX4 or WIF1 expression. All target sequences are listed in Table 1. Vectors and negative controls were simultaneously transfected into 293T cells using Lipofectamine 2000 (Invitrogen). Post‐transfection, the supernatant of the medium was collected, filtered through a 0.45 μm filter, and subsequently used to infect RCC cells with 12.5 μg/mL polybrene. Following a 72‐h transfection period, stably transfected cells were selected by subjecting them to treatment with 2 μg/mL puromycin.

**TABLE 1:** Target sequences.

Gene	Forward (5′ → 3′)	Reverse (5′ → 3′)
sh‐PRDX4	AAAUCUUCGCUUUGCUUAGGU	CUAAGCAAAGCGAAGAUUUCC
PRDX4	GCGAAGAUUUCCAAGCCAGC	AGUCUGUCGCCAAAAGCGAU
sh‐WIF1	AAUCCUAUGAGUACUCUUGCC	CAAGAGUACUCAUAGGAUUUG

#### 2.4.1 Cell Viability Assay

RCC cell viability was assessed using the CCK8 assay, according to the manufacturer's instructions. Briefly, 1 × 10^3^ cells were seeded in a 96‐well plate. After 24 h of incubation, the culture medium was replaced with a gradient of THSG concentrations. After additional 12, 24, and 48 h of culture, the supernatant was aspirated, and CCK8 (20 μL) was added to each well. Optical density (OD) was measured at 490 nm using a microplate reader.

#### 2.4.2 Cell Apoptosis Assay

The RCC cells were suspended in 100 μL of labeling solution (100 μL) and cultured at 37°C for 15 min. Subsequently, the fluorescence solution was introduced into the cells and incubated in the dark at 4°C for 20 min. Finally, a flow cytometer (Millipore, USA) was used to detect the fluorescence emitted by FITC at 515 nm and PI at 560 nm.

#### 2.4.3 Cell Invasion Assay

The inserts were coated with 20 μL of 1:2‐diluted Matrigel, and the coated inserts were then treated with varying concentrations of TSG for 24 h to induce matrix interactions. Following treatment, 1 × 10^5^ RCC cells were seeded into the upper chamber of the Matrigel‐coated inserts, which contained 100 μL of serum‐free medium to prevent cell proliferation from serum factors. After 24 h of incubation at 37°C, the lower chamber was filled with 500 μL of medium supplemented with 10% FBS to promote cell migration and chemotaxis. After the incubation period, non‐migratory cells on the upper surface of the filter were gently removed using a cotton swab. The filters were then stained with 0.1% crystal violet solution for 10 min. The stained cells were examined under an Olympus microscope (Tokyo, Japan), and images were captured to assess cell invasion.

#### 2.4.4 Wound Healing Assay

RCC cells were cultured in six‐well plates and maintained until they reached 90% confluence. The culture medium was then replaced with serum‐free DMEM to minimize the influence of serum on cell migration. Once the cells reached full confluence, a sterile scratch was made in the cell monolayers using a 100 μL sterile pipette tip, creating a uniform wound. The plates were then washed thoroughly with PBS to remove any detached cells and debris. After a 24‐h incubation period at 37°C with 5% CO_2_, the wound closure was observed and photographed using a Leica DM 14000 B optical microscope (Leica, Germany) at a suitable magnification.

#### 2.4.5 Quantitative PCR (qPCR)

Total RNA was extracted from RCC tissues and cells using TRIzol Reagent. Following this, cDNA was synthesized with the First‐Strand cDNA kit. Subsequently, qPCR was employed using the SYBR Green kit. The qPCR reaction was set for 40 cycles of 94°C for 5 min and 94°C for 20 s; 60°C for 1 min; collect signals at 60°C. In order to perform relative quantitative analysis, GAPDH was utilized as an internal reference. Primer sequences are depicted in Table 2.

**TABLE 2:** Primer sequence.

Gene	Forward (5′ → 3′)	Reverse (5′ → 3′)
PRDX4	GCGAAGATTTCCAAGCCAGC	AGTCTGTCGCCAAAAGCGAT
GAPDH	GGAGCGAGATCCCTCCAAAAT	GGCTGTTGTCATACTTCTCATGG

#### 2.4.6 Western Blotting

The protein was extracted by RIPA and PMSF. After using the BCA kit to measure protein concentration, the proteins were transferred to the PVDF membrane. Apply skim milk to block the membrane, dilute the first antibody to a designated volume, and incubate overnight at 4°C. Post‐washing, incubate the specific secondary antibody for 2 h and expose the target band in a chemiluminescent solution. The detection of protein bands was carried out with Prime Western Blotting Reagent (Cytiva, UK), and their gray values were analyzed with ImageJ. The antibodies used in this study were sourced from Abcam (UK).

#### 2.4.7 Co‐Immunoprecipitation (Co‐IP) Assay

CO‐ip assays were performed as described previously (Kocatürk 2023). Briefly, total protein was extracted from RCC cells using NP‐40 lysis buffer. Protein concentrations were determined using a BCA assay (or similar method) to quantify the total protein content. The protein samples were then divided into three aliquots: Input, IgG, and IP, with each containing approximately 1 mg of protein. To each aliquot, 20 μL of protein A/G beads was added, and the samples were incubated for 2 h at 4°C with gentle rotation to allow binding. Following this, either IgG or specific immunoprecipitation (IP) antibodies were added to the respective samples, and the mixture was incubated overnight at 4°C to facilitate antibody‐protein interactions. On the following day, an additional 20 μL of protein A/G beads was added to the samples, and they were incubated for another 2 h at 4°C with rotation. Magnetic beads were then collected by placing the samples on a magnetic rack, and the beads were washed to remove unbound proteins. The beads were resuspended in NP‐40 lysis buffer containing a loading buffer, and the proteins were denatured by heating at 95°C for 5 min. The supernatant, containing the denatured proteins, was collected via centrifugation. Finally, protein–protein interactions were assessed by western blot analysis, where the specific interactions were detected using appropriate primary and secondary antibodies.

#### 2.4.8 Animals

Eight male nude mice were used in the present study. The nude mice were provided with free access to food and water, while the temperature and relative humidity of the room were maintained at 20°C–24°C and 40%–60%, respectively. Eight mice were allowed to acclimate to their surroundings for 1 week prior to experimentation. Animal experiments adhered to institutional guidelines to ensure ethical conduct.

#### 2.4.9 In Vivo Studies

The A498 cell line was used to establish a tumor xenograft model in nude mice. Briefly, 1 × 10^7^ cells were injected into the right flank of nude mice. Seven days after cell injection, 10 mice were randomly assigned to either the control group (*n* = 5) or the resveratrol group (*n* = 5). Mice in the resveratrol group were administered resveratrol (50 mg/kg) daily for 25 consecutive days, whereas those in the control group were administered an equal volume of solvent (DMSO). Tumor volumes were measured using Vernier calipers at 5‐day intervals. At the conclusion of the experimental period, the mice were euthanized and tumor weights were determined.

#### 2.4.10 Immunohistochemistry

The paraffin sections were deparaffinized for antigen retrieval and washed three times with PBS for 5 min each. Subsequently, serum was used for blocking. The primary antibodies PCNA and Ki67 were applied and incubated at 4°C overnight. The following day, the sections were washed three times with PBS, each for 5 min. The diluted secondary antibody was added and incubated at room temperature for 50 min. Subsequently, the sections were washed thrice with PBS for 5 min each. The DAB color developing solution was carefully applied dropwise. After the color reaction, counterstaining with hematoxylin was performed, which shifted the hue back to blue. Subsequently, the sections were dehydrated and made transparent. After drying, the sections were observed under a microscope.

#### 2.4.11 Statistical Analysis

GraphPad Prism 9.0 software was used to analyze the data. Continuous data was represented using the format means ± standard deviation (*x* ± *s*). The *t*‐test was used for comparison between two groups. One‐way ANOVA was used to compare multiple groups. The KM Plotter online tool was used to generate survival analysis in patients with RCC (Györffy et al. 2010). A *p‐*value < 0.05 represented a statistically significant difference.

## 3 Results

### 3.1 Resveratrol Suppressed Malignant Biological Behavior of RCC Cells

**FIGURE 1:**
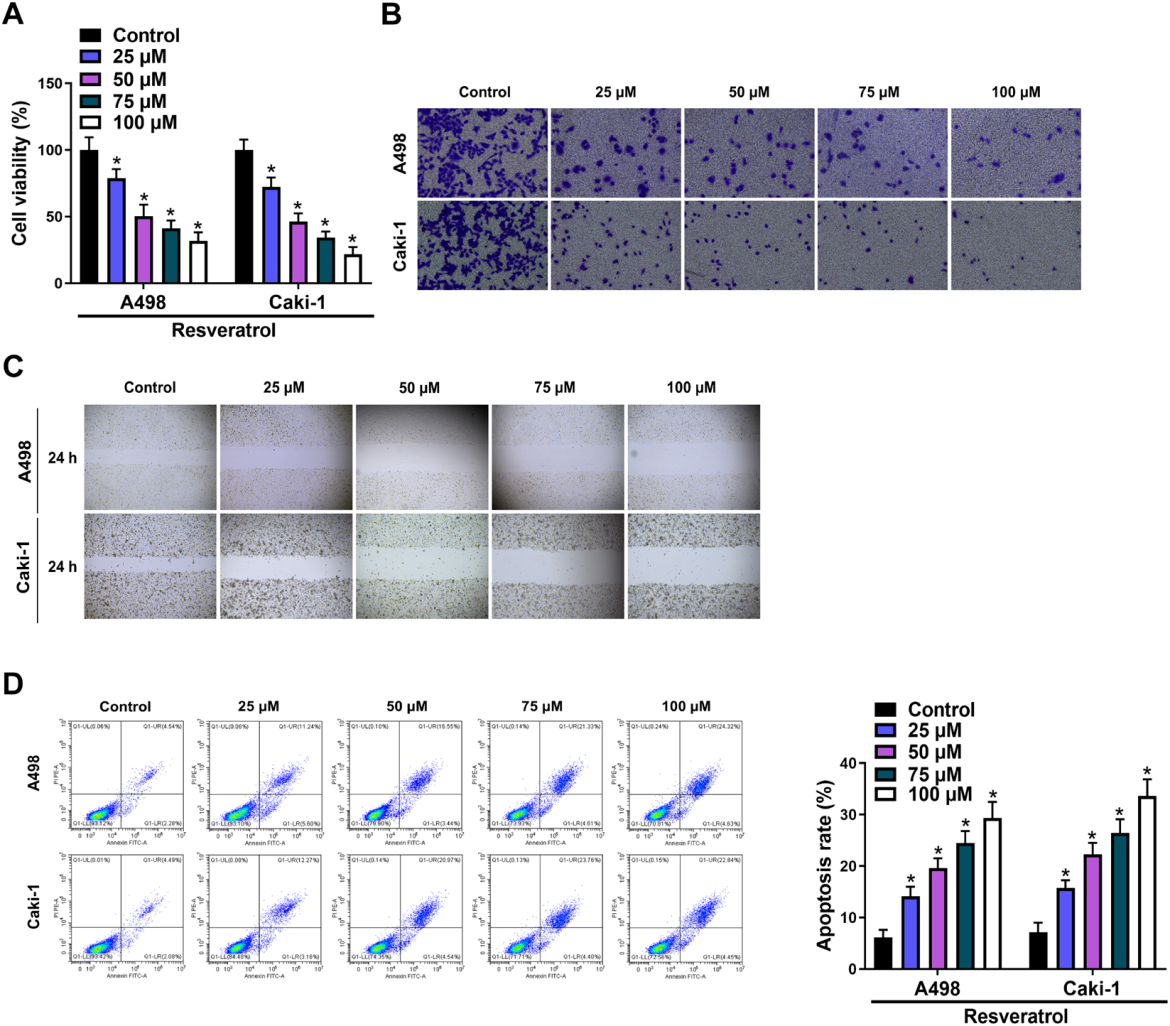
Resveratrol suppressed malignant biological behavior of RCC cells. (A) CCK8 assay was used to detect the changes in cell viability in A498 and Caki‐1 cells after treatment with different concentrations of resveratrol. (B) Transwell assay was used to detect the changes in cell invasion in A498 and Caki‐1 cells after treatment with different concentrations of resveratrol. (C) Wound healing assay was used to detect the changes in cell migration in A498 and Caki‐1 cells after treatment with different concentrations of resveratrol. (D) Flow cytometry was used to detect the changes in cell apoptosis in A498 and Caki‐1 cells after treatment with different concentrations of resveratrol. *p < 0.05 by one‐way ANOVA.

To investigate the effects of resveratrol on RCC, A498, and Caki‐1 cells were subjected to varying concentrations of resveratrol. Our results revealed that resveratrol inhibited A498 and Caki‐1 cell viability in concentration‐dependent manners (Figure 1A). Moreover, Transwell and wound healing assays demonstrated that resveratrol significantly suppressed cell metastasis in A498 and Caki‐1 cells (Figure 1B,C). Furthermore, flow cytometry analysis showed that resveratrol notably increased apoptosis in A498 and Caki‐1 cells in a dose‐dependent manner (Figure 1E). Given that the maximum effectiveness of resveratrol against cancer was observed at 100 μM, this concentration was selected for subsequent experiments.

### 3.2 Resveratrol Inhibited RCC Tumor Growth In Vivo

**FIGURE 2:**
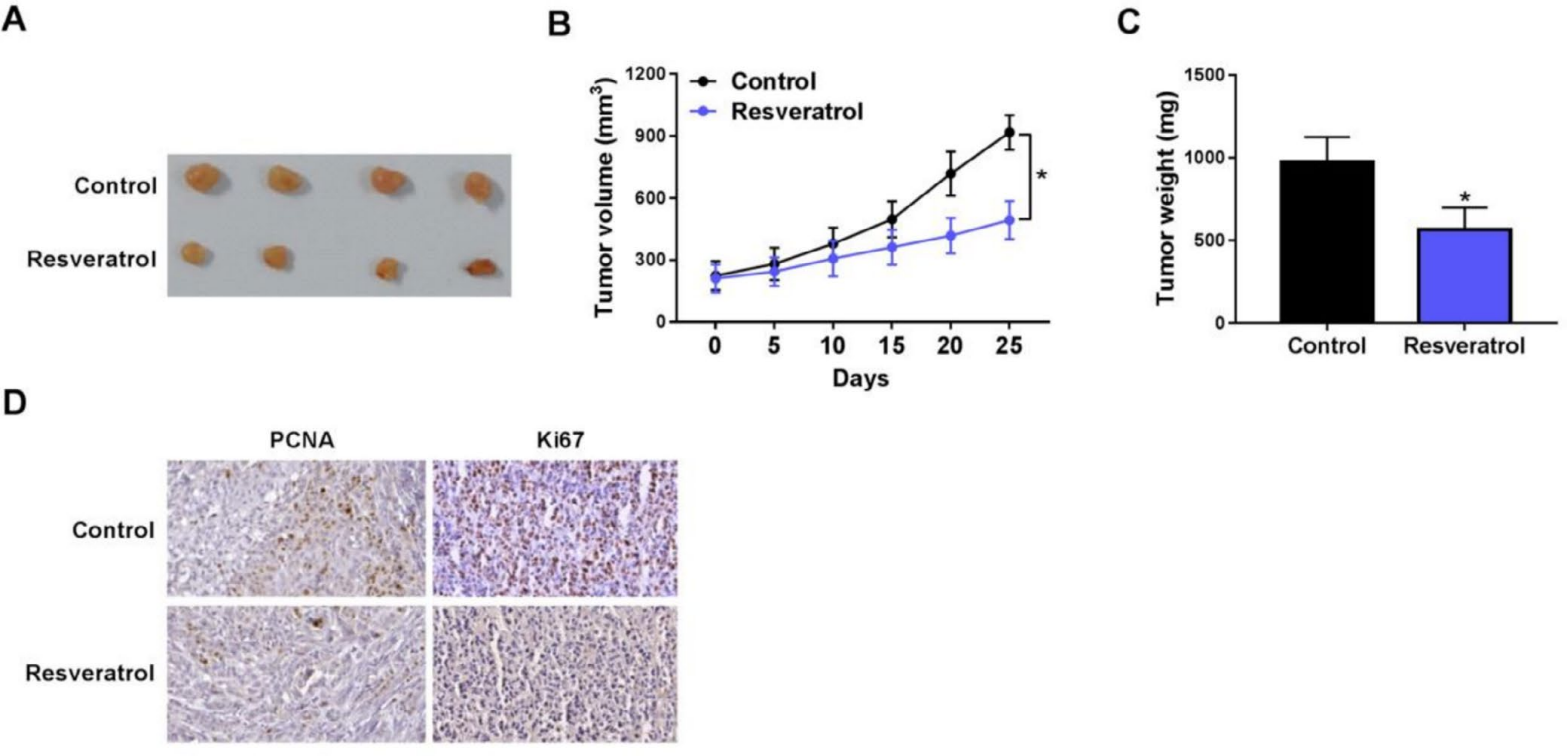
Resveratrol inhibited RCC tumor growth in vivo. (A) Representative images of tumor tissues from each group. (B) Tumor volumes measured on various days following resveratrol injection in each group (C) The tumor weight change of mice following resveratrol injection. (D) Changes in PCNA and Ki67 protein expression in tumor tissues following resveratrol injection. *p < 0.05 by *t* test (A, B and E–H) and one‐way ANOVA (C, D).

To assess the influence of resveratrol on RCC tumor growth, xenograft tumor models were established, and resveratrol was subsequently administered to mice via gavage. Representative images of the tumors are presented in Figure 2A. Compared with the control group, tumor volume and weight were significantly reduced in the resveratrol group (Figure 2B,C). Subsequently, we examined PCNA and ki67 expression in RCC tumor tissues using immunohistochemical analysis. Results indicated a notable reduction in PCNA and ki67 expressions in tumor tissues after resveratrol treatment (Figure 2D).

### 3.3 Influences of PRDX4 on the Biological Behavior in RCC Cells

**FIGURE 3:**
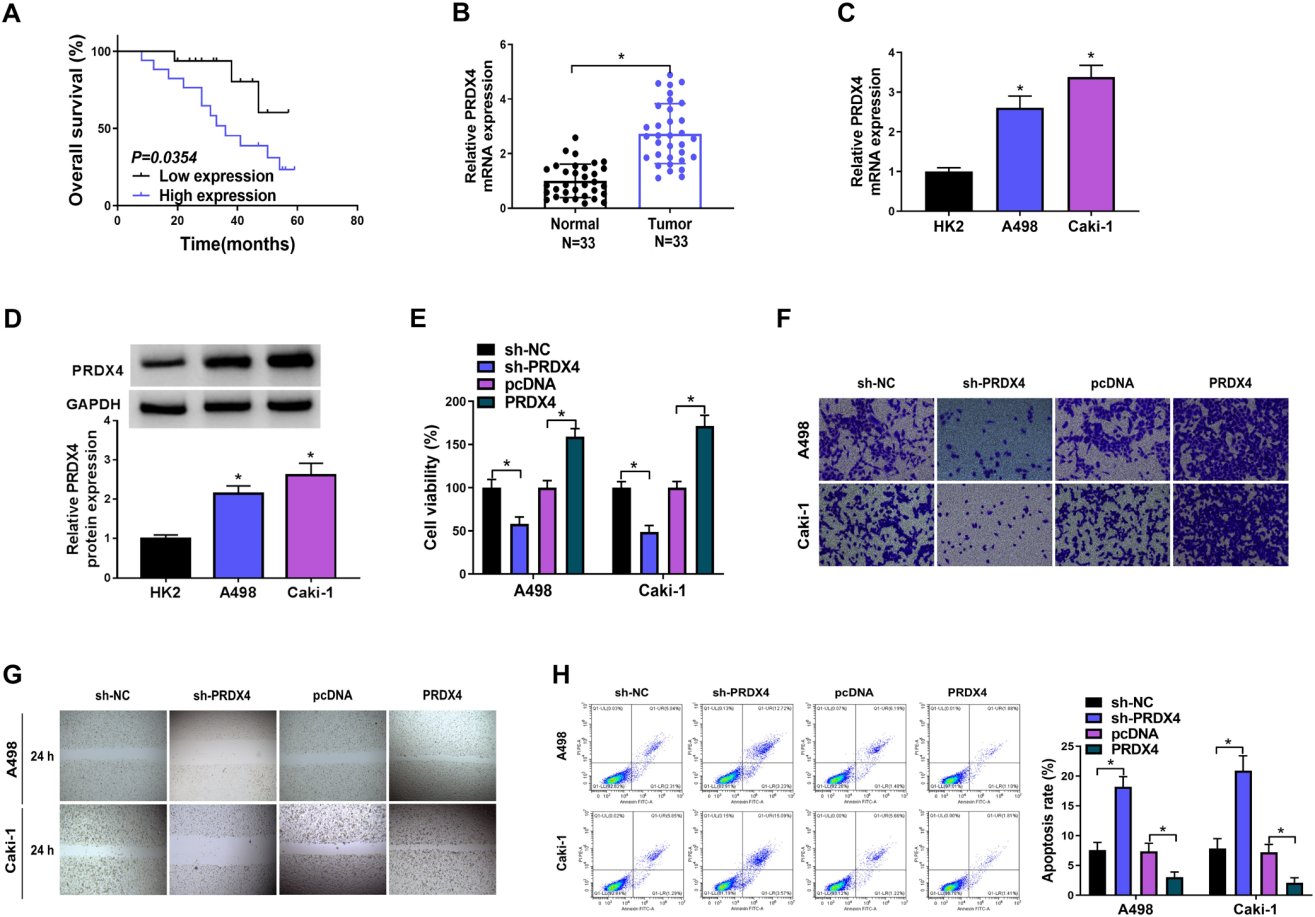
The effects of PRDX4 on malignant biological behavior of RCC cells. (A) Survival curves of patients with low‐ and high‐expression of PRDX4. (B) The PRDX4 protein expression in RCC tissues. (C) The PRDX4 mRNA levels in HK2, A498, and Caki‐1 cells were detected by qPCR. (D) The PRDX4 protein expression in HK2, A498, and Caki‐1 cells was detected by western blot. (E) CCK8 assay was used to detect the changes in cell viability in A498 and Caki‐1 cells upon PRDX4 silencing or overexpression. (F) Transwell assay was used to detect the changes in cell invasion in A498 and Caki‐1 cells upon PRDX4 silencing or overexpression. (G) Wound healing assay was used to detect the changes in cell migration in A498 and Caki‐1 cells upon PRDX4 silencing or overexpression (H) Flow cytometry was used to detect the changes in cell apoptosis in A498 and Caki‐1 cells upon PRDX4 silencing or overexpression. *p < 0.05 by *t* test. Full western blot images can be found at Supporting Information.

Previous studies have reported an upregulation of PRDX4 in various human tumors. The KM Plotter online database revealed that RCC patients with elevated PRDX4 expression experienced significantly shorter survival times (Figure 3A). This observation prompted us to hypothesize that PRDX4 plays a role in the progression of RCC. PRDX4 expression was significantly higher in RCC tissues than in noncancerous tissues (Figure 3B). Moreover, both mRNA and protein levels were notably increased in A498 and Caki‐1 cells compared with those in HK‐2 cells (Figure 3C,D). To investigate the effects of PRDX4 on the biological functions of RCC cells, we transfected sh‐PRDX4 or PRDX4 into A498 and Caki‐1 cells. The CCK8 assay showed that PRDX4 silencing significantly inhibited the viability of A498 and Caki‐1 cells, while PRDX4 overexpression notably increased cell viability (Figure 3E). Additionally, PRDX4 knockdown notably suppressed the A498 and Caki‐1 cell metastasis, whereas PRDX4 overexpressing significantly enhanced cell metastasis (Figure 3F,G). Furthermore, silencing PRDX4 significantly increased apoptosis inA498 and Caki‐1 cells. In contrast, PRDX4 overexpression decreased apoptosis of A498 and Caki‐1 cells (Figure 3H).

### 3.4 Resveratrol Suppressed Malignant Biological Behavior of RCC Cells by Downregulating PRDX4

**FIGURE 4:**
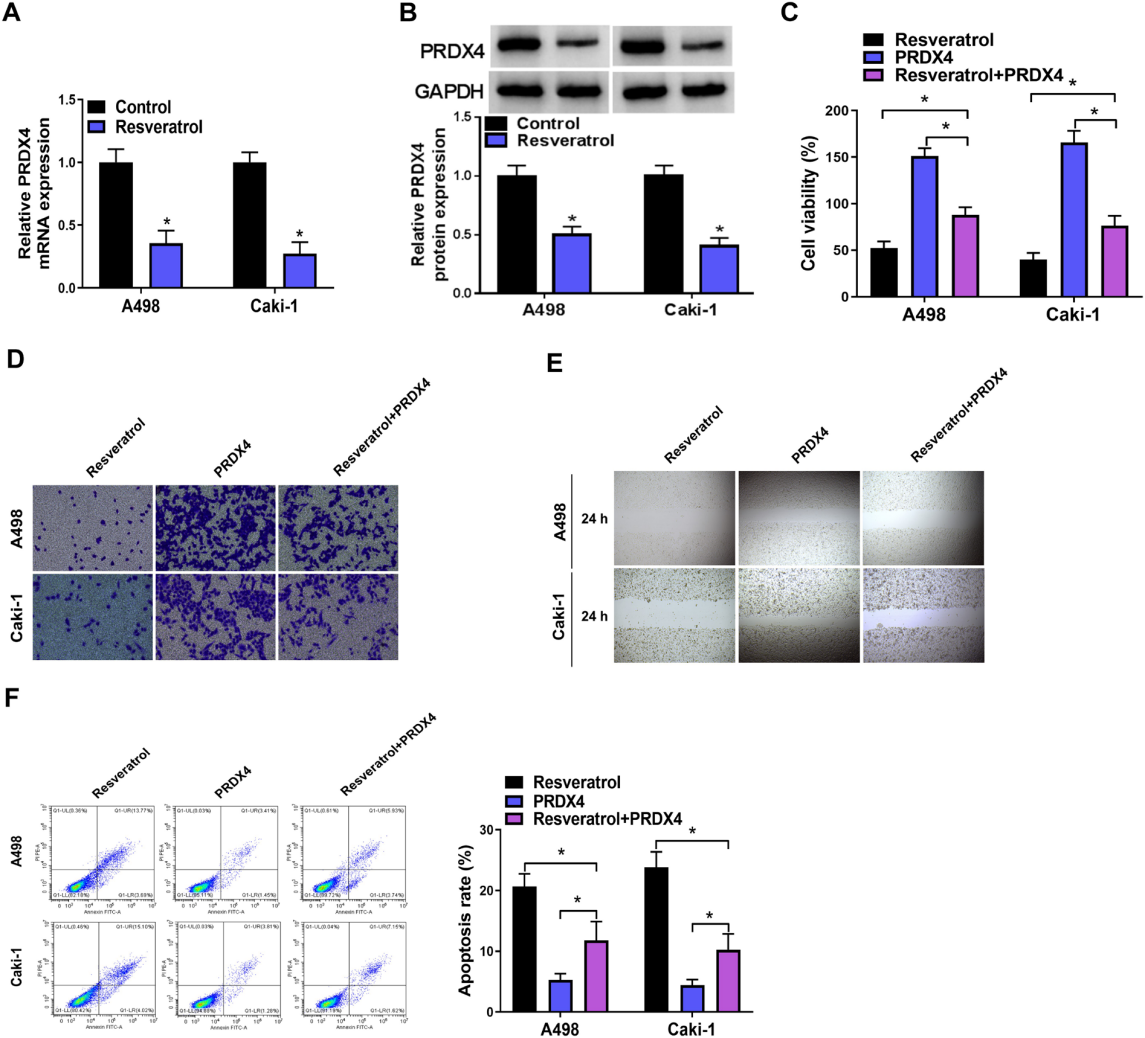
Resveratrol suppressed malignant biological behavior of RCC cells by downregulating PRDX4. (A) qPCR was used to detect the changes in PRDX4 mRNA levels in PRDX4‐overexpressing A498 and Caki‐1 cells after treatment with 100 μM resveratrol. (B) Western blot was used to detect the changes in PRDX4 protein expression in PRDX4‐overexpressing A498 and Caki‐1 cells after treatment with 100 μM resveratrol. (C) CCK8 assay was used to detect the changes in cell viability in PRDX4‐overexpressing A498 and Caki‐1 cells after treatment with 100 μM resveratrol (D) Transwell assay was used to detect the changes in cell invasion in PRDX4‐overexpressing A498 and Caki‐1 cells after treatment with 100 μM resveratrol. (E) Wound healing assay was used to detect the changes in cell migration in PRDX4‐overexpressing A498 and Caki‐1 cells after treatment with 100 μM resveratrol. (F) Flow cytometry was used to detect the changes in cell apoptosis in PRDX4‐overexpressing A498 and Caki‐1 cells after treatment with 100 μM resveratrol. *p < 0.05 by *t* test (A, B) and one‐way ANOVA (C–F). Full western blot images can be found in Supporting Informations.

Next, we investigated whether resveratrol influenced the expression of PRDX4 in RCC cells. Results from qPCR and Western blot analyses demonstrated a significant downregulation of PRDX4 in A498 and Caki‐1 cells treated with resveratrol (Figure 4A,B). Subsequently, we performed rescue experiments by treating PRDX4‐overexpressing RCC cells with resveratrol. The findings revealed that PRDX4 overexpression alleviated the inhibitory effects of resveratrol on A498 and Caki‐1 cell viability and metastasis (Figure 4C–E). Furthermore, PRDX4 overexpression reversed the pro‐apoptotic effects induced by resveratrol (Figure 4F).

### 3.5 Resveratrol Inhibited the Wnt/β‐Catenin Signaling Pathway in RCC Cells

**FIGURE 5:**
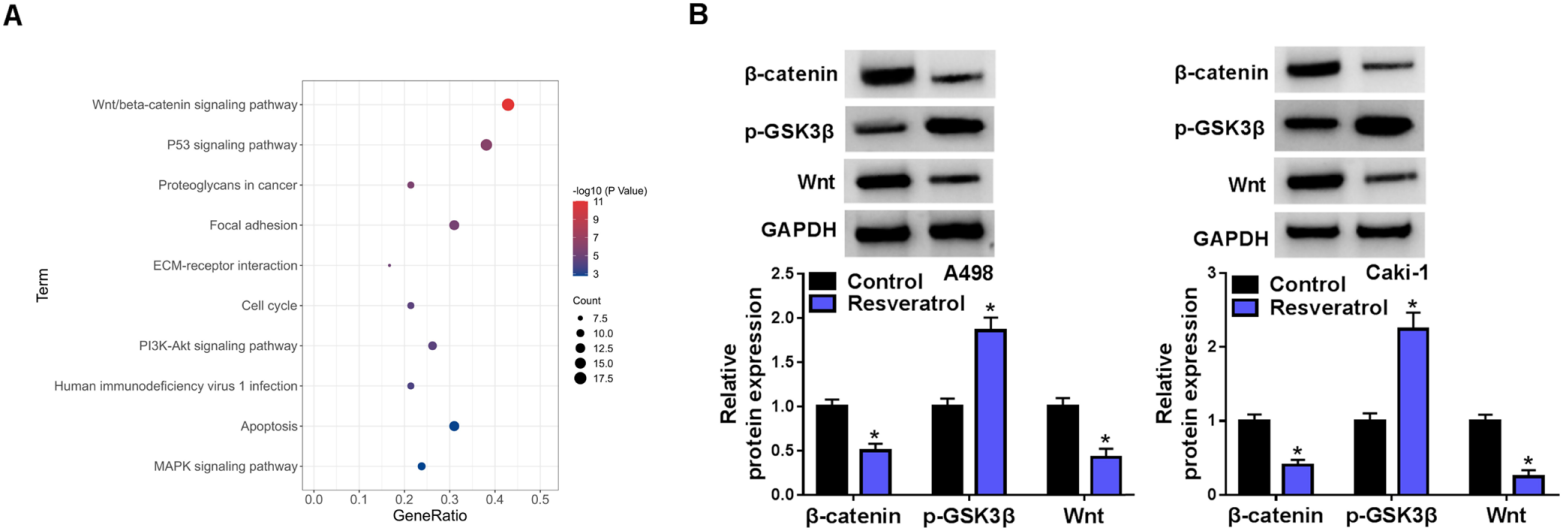
Resveratrol inhibited the Wnt/β‐catenin pathway in RCC cells. (A) Dotplot of KEGG pathway analysis. (B) Western blot was used to detect the changes in β‐catenin, p‐GSK3B, and Wnt protein expression in A498 and Caki‐1 cells after treatment with 100 μM resveratrol. *p < 0.05 by *t* test.

To explore the potential molecular mechanisms underlying RCC progression, we conducted a bioinformatics analysis. KEGG pathway analysis revealed that the Wnt/β‐catenin signaling pathway was the most significantly enriched pathway (Figure 5A). To validate the bioinformatic analysis results, Western blot assay was performed. The findings indicated that resveratrol significantly reduced β‐catenin and Wnt expression while enhancing p‐GSK3B expression (Figure 5B).

### 3.6 Resveratrol Inhibited Malignant Biological Behavior of RCC Cells via Wnt/β‐Catenin Pathway

**FIGURE 6:**
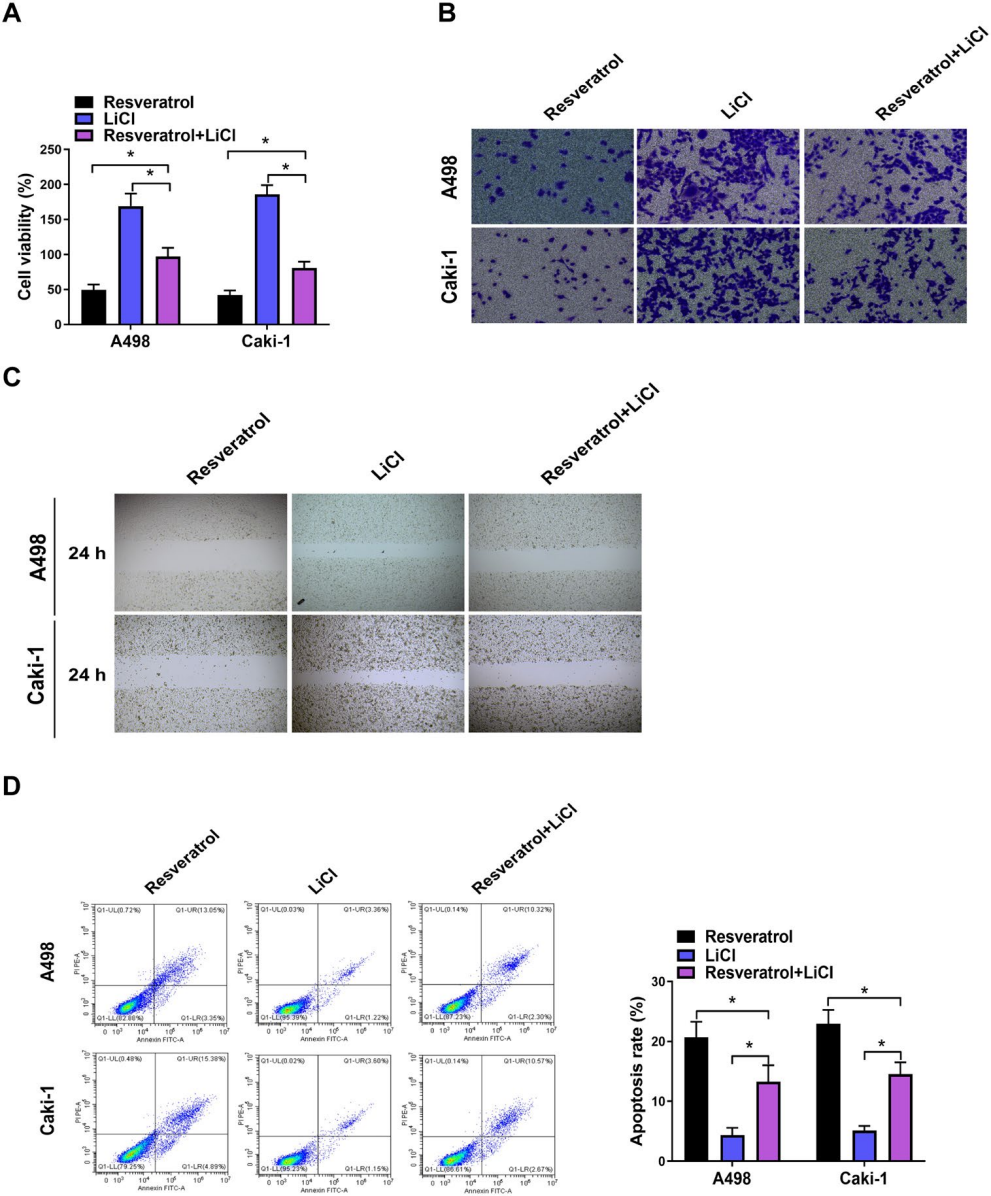
Resveratrol suppressed malignant biological behavior of RCC cells through the Wnt/β‐catenin pathway. (A) CCK8 assay was used to detect the changes in cell viability in A498 and Caki‐1 cells after treatment with 100 μM resveratrol and LiCl simultaneously. (B) Transwell assay was used to detect the changes in cell invasion in A498 and Caki‐1 cells after treatment with 100 μM resveratrol and LiCl simultaneously. (C) Wound healing assay was used to detect the changes in cell migration in A498 and Caki‐1 cells after treatment with 100 μM resveratrol and LiCl simultaneously. (D) Flow cytometry was used to detect the changes in cell apoptosis in A498 and Caki‐1 cells after treatment with 100 μM resveratrol and LiCl simultaneously. *p < 0.05 by one‐way ANOVA.

RCC cells were next subjected to treatment with LiCl, which is an activator of Wnt/β‐catenin pathway (Ning et al. 2012). The results showed that LiCl mitigated the inhibitory impact of resveratrol on A498 and Caki‐1 cell viability and metastasis (Figure 6A–C). Additionally, LiCl reversed the pro‐apoptotic effects of resveratrol in A498 and Caki‐1 cells (Figure 6E).

### 3.7 PRDX4 Knockdown Suppressed Malignant Biological Behavior of RCC Cells via Wnt/β‐Catenin Pathway

**FIGURE 7:**
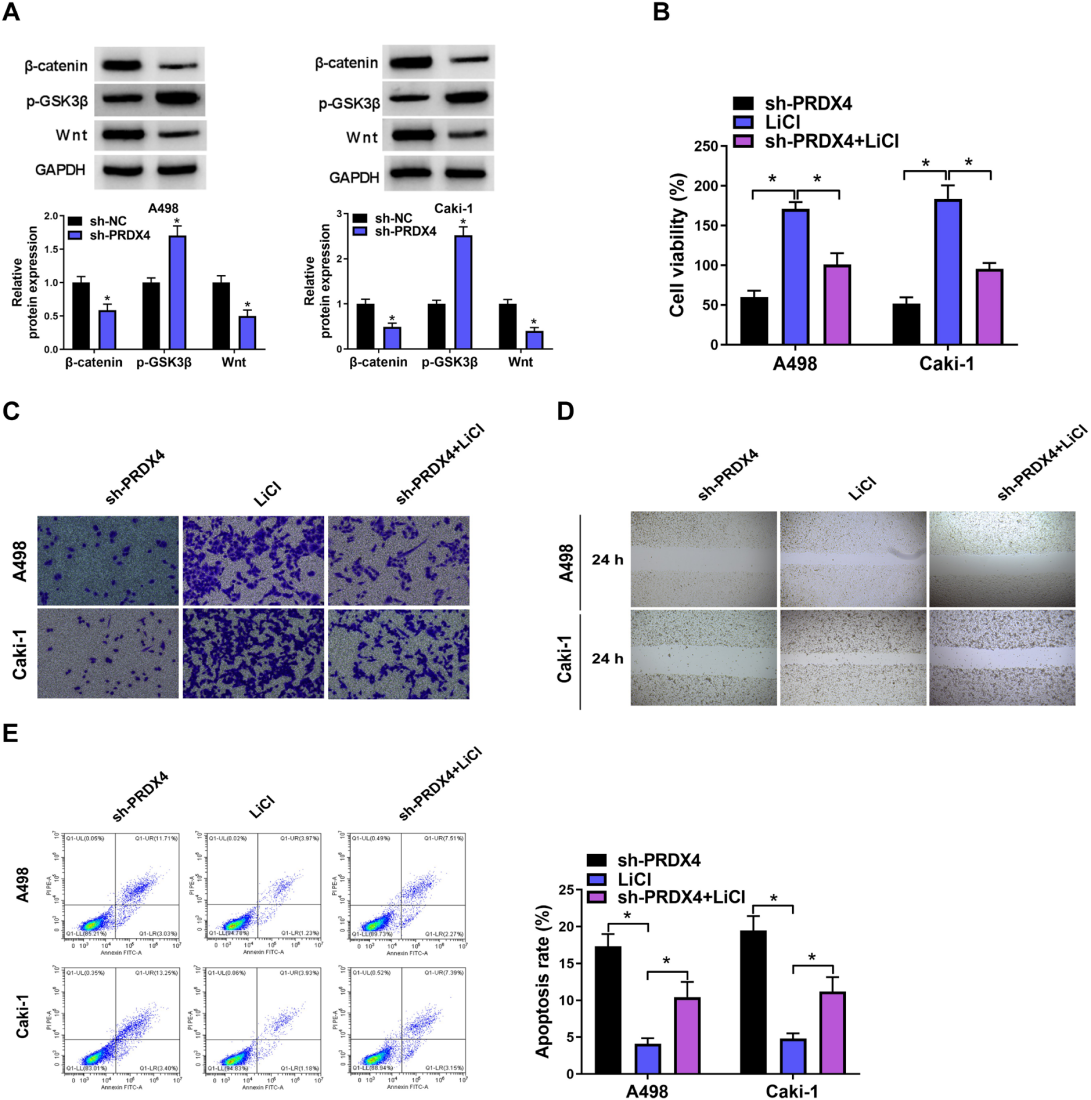
PRDX4 silencing inhibited malignant biological behavior of RCC cells through the Wnt/β‐catenin pathway. (A) Western blot was used to detect the changes in β‐catenin, p‐GSK3B, and Wnt protein expression in A498 and Caki‐1 cells upon PRDX4 silencing. (B) CCK8 assay was used to detect the changes in cell viability in PRDX4‐silenced A498 and Caki‐1 cells after treatment with LiCl (C) Transwell assay was used to detect the changes in cell invasion in PRDX4‐silenced A498 and Caki‐1 cells after treatment with LiCl. (D) Wound healing assay was used to detect the changes in cell migration in PRDX4‐silenced A498 and Caki‐1 cells after treatment with LiCl. (E) Flow cytometry was used to detect the changes in cell apoptosis in PRDX4‐silenced A498 and Caki‐1 cells after treatment with LiCl. *p < 0.05 by t test (A) one‐way ANOVA (B–E). Full Western blot images can be found in Supporting Information.

We employed western blot analysis to investigate the influence of PRDX4 on Wnt/β‐catenin pathway. As shown in Figure 7A, the Wnt/β‐catenin pathway was significantly inhibited following PRDX4 silencing. Subsequently, PRDX4‐silenced A498 and Caki‐1 cells were subjected to LiCl treatment. The results indicated that LiCl alleviated the inhibitory impact of PRDX4 knockdown on A498 and Caki‐1 cell viability and metastasis (Figure 7B–D). Additionally, LiCl reversed the pro‐apoptotic effects induced by PRDX4 silencing in A498 and Caki‐1 cells (Figure 7E).

### 3.8 PRDX4 Silencing Inhibited Malignant Biological Behavior of RCC Cells by Increasing WIF1 Transcription

**FIGURE 8:**
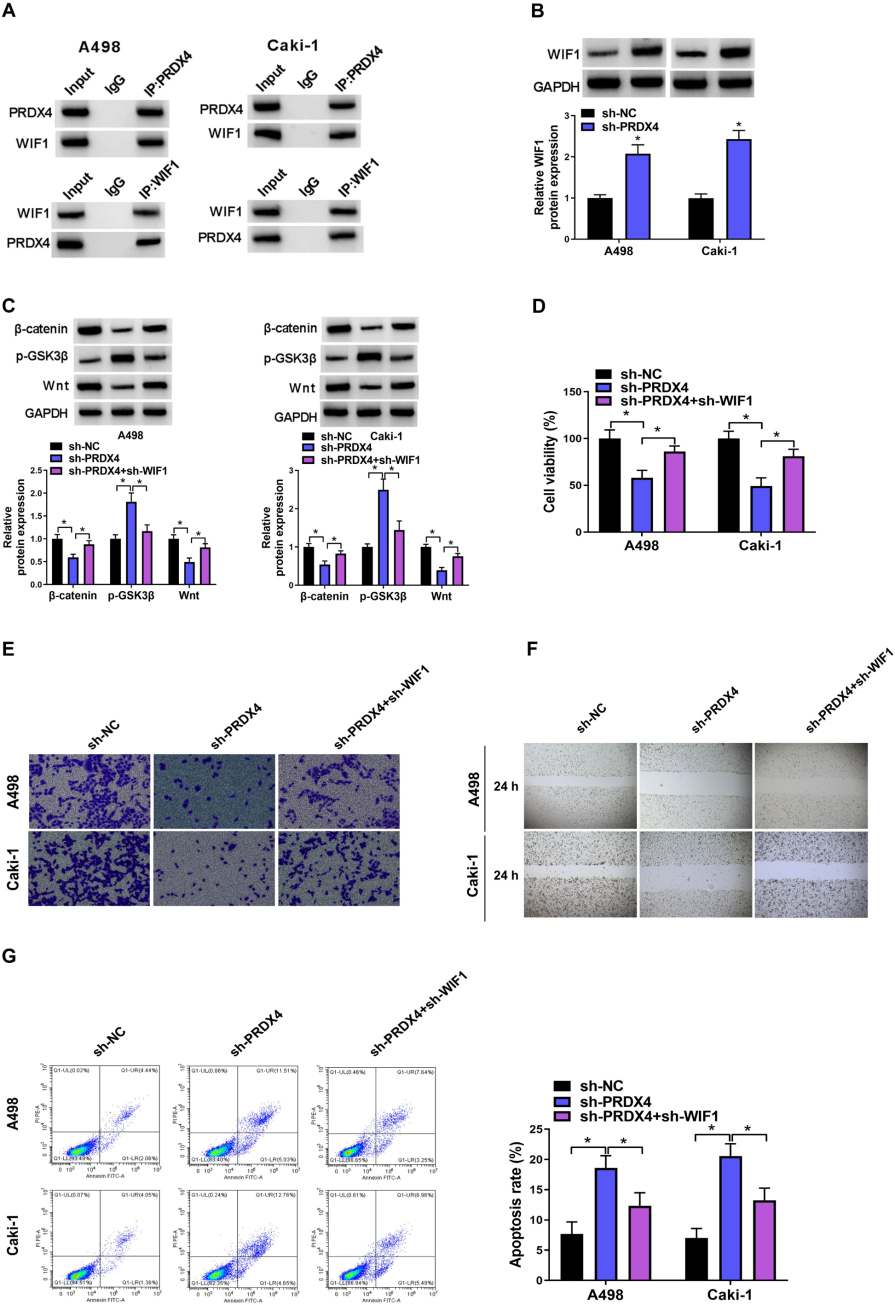
PRDX4 silencing inhibited malignant biological behavior of RCC cells by increasing WIF1 transcription. (A) PRDX4 and WIF1 could bind with each other in A498 and Caki‐1 cells. (B) Western blot was used to detect the changes in WIF1 protein expression in A498 and Caki‐1 cells upon PRDX4 silencing. (C) Western blot was used to detect the changes in β‐catenin, p‐GSK3B, and Wnt protein expression in PRDX4‐silenced A498 and Caki‐1 cells upon WIF1 silencing. (D) CCK8 assay was used to detect the changes in cell viability in PRDX4‐silenced A498 and Caki‐1 cells upon WIF1 silencing. (E) Transwell assay was used to detect the changes in cell invasion in PRDX4‐silenced A498 and Caki‐1 cells upon WIF1 silencing. (F) Wound healing assay was used to detect the changes in cell migration in PRDX4‐silenced A498 and Caki‐1 cells upon WIF1 silencing. (G) Flow cytometry was used to detect the changes in cell apoptosis in PRDX4‐silenced A498 and Caki‐1 cells upon WIF1 silencing. *p < 0.05 by *t* test (B) one‐way ANOVA (C–G). Full western blot images can be found in the Supporting Informations.

WIF1 functions as a secreted Wnt inhibitor, directly binding to Wnt and preventing the interaction of Wnt ligands with its receptors (Zhang et al. 2022). In A498 and Caki‐1 cells Co‐IP results revealed that PRDX4 could interact with WIF1 to form a protein–protein complex (Figure 8A). Western blot analysis further revealed that the PRDX4 silencing notably enhanced WIF1 expression (Figure 8B). Subsequently, WIF1 was downregulated by introducing a sh‐WIF1 plasmid. Compared to RCC cells transfected with sh‐PRDX4 alone, co‐transfection with sh‐PRDX4 and sh‐WIF1 promoted the activation of the Wnt/β‐catenin signaling pathway (Figure 8C). Moreover, WIF1 knockdown remarkedly reversed the anticancer effects induced by PRDX4 silencing in A498 and Caki‐1 cells (Figure 8D–G).

### 3.9 Resveratrol Inhibited the Wnt/β‐Catenin Pathway by Downregulating PRDX4 in RCC Cells

**FIGURE 9:**
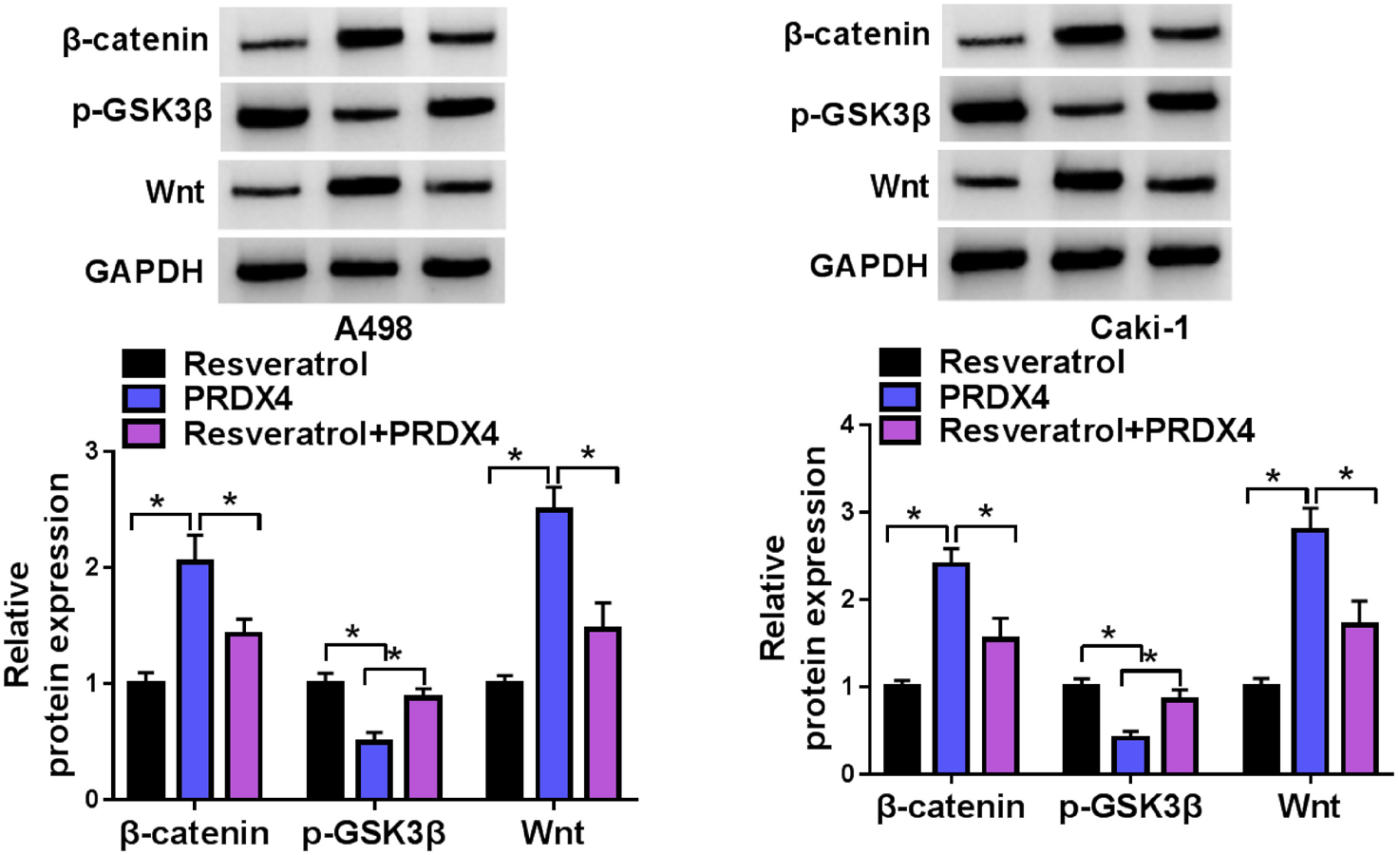
Resveratrol inhibited the Wnt/β‐catenin pathway via downregulating PRDX4 in RCC cells. *p < 0.05 by one‐way ANOVA. Full western blot images can be found at Supporting Information.

Finally, western blot analysis was conducted to substantiate the suppression of the Wnt/β‐catenin signaling pathway by resveratrol through the regulation of PRDX4 expression. The results indicated that resveratrol significantly diminished the activation of the Wnt/β‐catenin signaling pathway in A498 and Caki‐1 cells. Nevertheless, the transfection of PRDX4 overexpression plasmid effectively reversed this aforementioned effect (Figure 9).

## 4 Discussion

Naturally occurring compounds for cancer treatment have recently garnered increasing attention. Resveratrol, a natural polyphenol compound, exhibits notable antitumor effects across various types of malignancies (Li et al. 2014; Zhong et al. 2016; Park et al. 2016). Notably, previous research has shown that resveratrol can induce cell cycle arrest in the S phase, thereby inducing apoptosis in breast cancer 4T1 cells (Wu et al. 2019). In a separate investigation, it was discovered that resveratrol repressed miR‐21 expression, consequently impeding the advancement of colorectal cancer through regulating p53 and AKT (Prasad and Bondy 2017). Furthermore, resveratrol demonstrated its efficacy in blocking tumor malignancy by inhibiting kinase function (Clark et al. 2017). However, the precise role of resveratrol in RCC progression was still unclear.

In this research, RCC cell lines A498 and Caki‐1 underwent treatment with varying concentrations of resveratrol to ascertain the most suitable doses. Our experimental outcomes revealed that resveratrol at 100 μM obviously suppressed cell viability and metastasis, while promoting apoptosis in RCC cells. Consequently, this concentration was selected for subsequent experiments. To confirm these in vitro findings, we performed in vivo experiments using nude mice. Given the extensive metabolism of resveratrol in the liver and intestines, its oral bioavailability is < 1% (Shu et al. 2015), necessitating the determination of an appropriate dosage for in vivo studies. A prior investigation revealed that continuous gavage of resveratrol (60 mg/kg) for 40 days significantly inhibited RCC tumor growth in mice (Tian et al. 2020). However, concerns have been raised regarding potential side effects of high doses, such as nephrotoxicity (Crowell et al. 2004). As a result, we conducted a preliminary experiment (data not shown) and found that continuous gavage of a lower dose (50 mg/kg) for 25 days effectively inhibited RCC tumor growth without causing mortality. Based on these findings, we selected this regimen for our in vivo experiments. Our results demonstrate that, compared to the control group, tumor volume and weight were significantly reduced in the resveratrol‐treated mice. In summary, these findings support the effectiveness of resveratrol in impeding the progression of RCC.

A 2023 publication found that PRDX4 was upregulated in RCC cells (Kocatürk 2023). PRDX4 is an antioxidant enzyme situated in the endoplasmic reticulum (Wang et al. 2019), exhibits heightened expression in various malignant tumors (Zhao et al. 2022). Interestingly, the anti‐carcinogenic effects of resveratrol are mediated by antioxidant mechanisms (Ren et al. 2021). In this study, we found resveratrol decreased PRDX4 expression in RCC cells. Therefore, our focus shifted to mining the influence of PRDX4 on RCC progression. qPCR and Western blot results revealed a notable elevation in PRDX4 expression in RCC tissue samples and cell lines. Additionally, patients with elevated PRDX4 expression experienced significantly shorter survival times. To elucidate the specific function of PRDX4 in RCC, we generated stable PRDX4‐overexpressing and PRDX4‐silenced RCC cells. These results unequivocally showed that PRDX4 silencing significantly attenuated viability and metastasis, while increasing apoptosis in RCC cells. However, PRDX4 overexpression exhibited opposite effects, indicating its role as a cancer‐promoting gene in RCC. To explore whether resveratrol could inhibit RCC progression by regulating PRDX4 expression, RCC cells transfected with the PRDX4‐overexpression plasmid were treated with resveratrol. Our findings indicated that PRDX4 overexpression partially reversed the inhibitory effect of resveratrol on RCC cells development, indicating resveratrol suppressed RCC progression via regulating PRDX4.

We subsequently conducted KEGG pathway analysis to investigate the molecular mechanisms associated with RCC development. Our findings revealed that the Wnt/β‐catenin signaling pathway was the most significantly enriched pathway. Wnt/β‐catenin pathway is essential for embryonic development and tissue homeostasis. The activation of Wnt/β‐catenin pathway instigated by Wnt ligands, initiating the suppression of β‐catenin degradation orchestrated by the destruction complex (Clevers 2006). Consequently, stabilized β‐catenin translocate to the nucleus, where it initiates the transcription of downstream genes. Dysregulation of Wnt/β‐catenin pathway has been implicated in various malignant tumors (Marson et al. 2008). Previous studies have demonstrated that resveratrol can impede the invasion and motility of colorectal cancer cells by targeting the Wnt/β‐catenin pathway (Zang et al. 2015; Ji et al. 2013). In this investigation, we found resveratrol suppressed the Wnt/β‐catenin pathway in RCC cells. Consistent outcomes were noted in RCC cells transfected with sh‐PRDX4. Furthermore, co‐IP results indicated that PRDX4 could form a protein–protein complex with secreted Wnt inhibitor WIF1. Previous studies found that WIF1 could bind to Wnt directly and prevent the interaction of Wnt ligands with its receptors (Santos et al. 2022). Building upon these findings, we formulated the hypothesis that resveratrol suppresses RCC progression by inhibiting Wnt/β‐catenin pathway through downregulating PRDX4. To validate this hypothesis, rescue experiments were conducted using LiCl, which is an activator for Wnt/β‐catenin s pathway. Our findings showed that LiCl not only negated the suppressive effect of resveratrol on RCC progression but also opposed the inhibitory consequences of PRDX4 silencing on RCC progression.

Lastly, Western blot analysis was performed to confirm whether resveratrol inhibits the Wnt/β‐catenin pathway by modulating PRDX4 in RCC cells. The results indicated that PRDX4 overexpression counteracted the resveratrol‐mediated suppression of the Wnt/β‐catenin pathway.

This study reveals the anti‐tumor activity of resveratrol in RCC. The potential molecular mechanism involves the inhibition of PRDX4 expression by resveratrol, which reduces its binding to the Wnt inhibitor WIF1. This disruption prevents the interaction between Wnt ligands and their receptors, inhibiting Wnt/β‐catenin pathway activation and subsequently suppressing RCC progression in vitro and in vivo. However, this study had some limitations. First, research on the potential side effects of oral administration of resveratrol is insufficient. Second, the comparative effectiveness of resveratrol and established cancer therapies remains underexplored. Finally, in vivo studies on resveratrol are limited. Future research should focus on comprehensive in vivo evaluations to further substantiate the clinical application of resveratrol and ensure its safety and efficacy in therapeutic settings.

## 5 Conclusion

**FIGURE 10:**
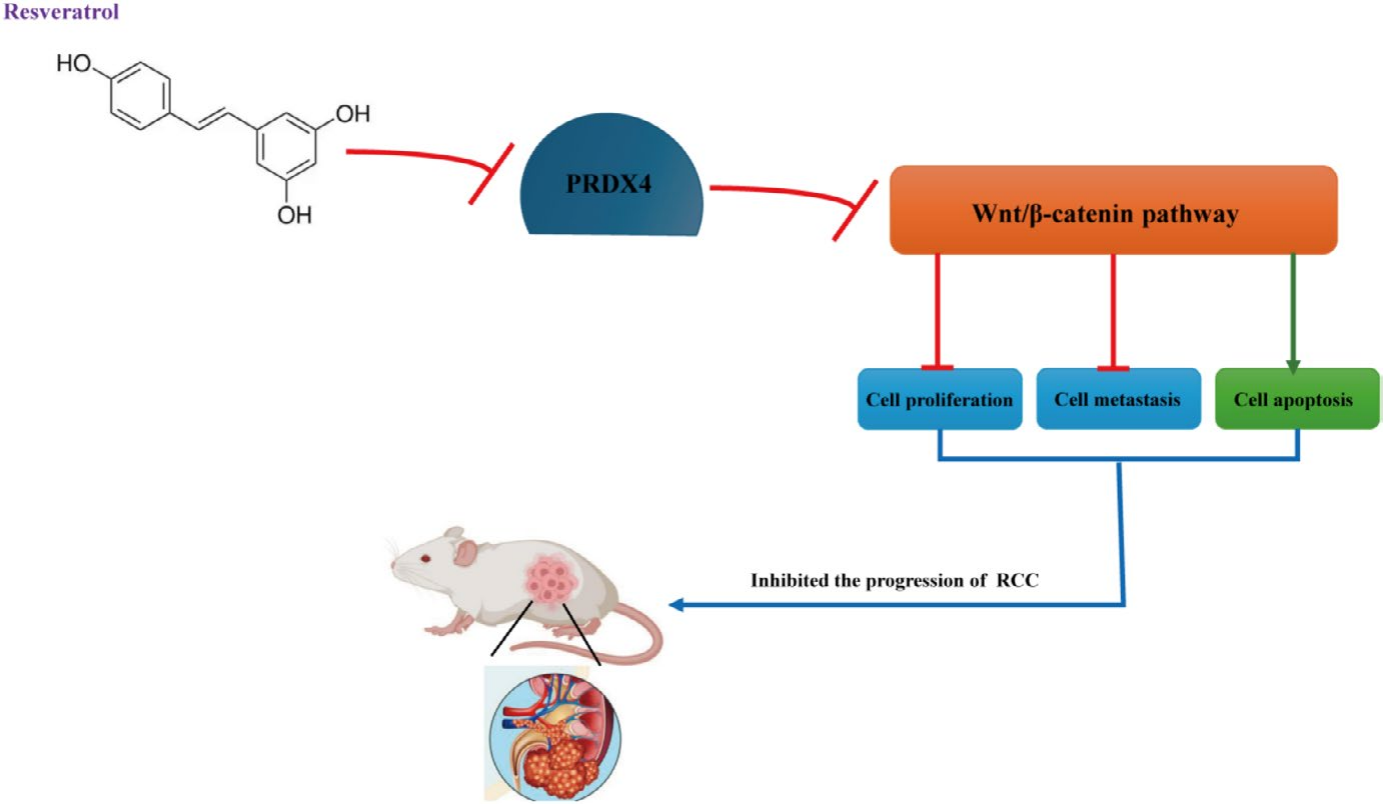
Mechanism diagram of resveratrol inhibiting RCC progression.

In summary, our investigations have validated the inhibitory impact of resveratrol on RCC progression. Mechanistic studies have shown that resveratrol suppresses the Wnt/β‐catenin pathway by decreasing PRDX4, thus inhibiting the RCC cell proliferation and metastasis and enhancing apoptosis (Figure 10). These findings suggest that resveratrol holds promise as a therapeutic agent for RCC treatment, and PRDX4 emerges as an innovative biomarker for RCC.

## Author Contributions

**Hongzhi Li:** conceptualization (equal), funding acquisition (equal), investigation (equal), methodology (equal), supervision (equal), validation (equal), visualization (equal), writing – original draft (equal), writing – review and editing (equal). **Zhun Wang:** data curation (equal), formal analysis (equal). **Xueji Chen:** investigation (equal), methodology (equal). **Shuai Li:** conceptualization (equal), investigation (equal), methodology (equal). **Fang Zhang:** investigation (equal), methodology (equal).

## Ethics Statement

The research was carried out following the principles of the Declaration of Helsinki and was approved by the Ethics Committee of Cangzhou Central Hospital (No. 2023‐376‐02) on January 22, 2024. All procedures were performed in compliance with relevant laws and institutional guidelines. The privacy rights of all participants have been observed. Signed informed consent were also obtained from all participants or guardians. Animal experiments was approved by the Experimental Animal Ethics Committee of Cangzhou Central Hospital (CZZX2023‐102‐01). Each animal received humane care. All animal experiments have complied with ARRIVE guidelines.

## Consent

Consent for publication was obtained from the patient.

## Conflicts of Interest

The authors declare no conflicts of interest.

## Supporting information


Supporting Information.

## Data Availability

Data will be available on reasonable request.
