# Supplementary material for: Resveratrol Downregulated PRDX4 Expression to Inhibit the Progression of Renal Cell Carcinoma via Wnt/β‐Catenin Pathway
Source: Food Sci Nutr. 2025 Jun 1;13(6):e70352. doi: 10.1002/fsn3.70352 (PMC12127141; doi:10.1002/fsn3.70352)
Supplement: Supplementary file 1 — Supporting Information. [file FSN3-13-e70352-s001.pdf]

The riginal western blots of Fig 3D

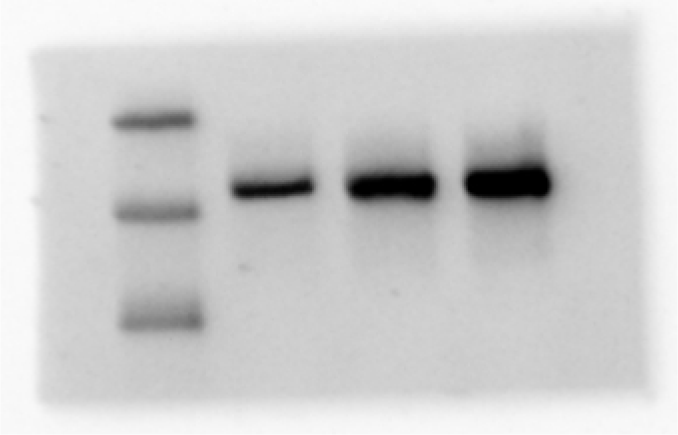

PRDX4

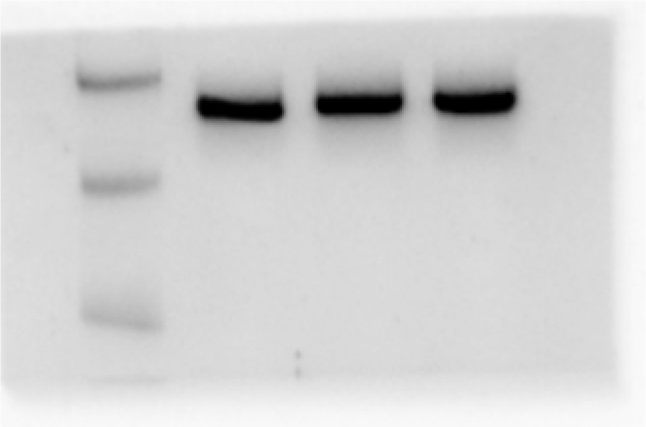

GAPDH

The original western blots of Fig4B

A

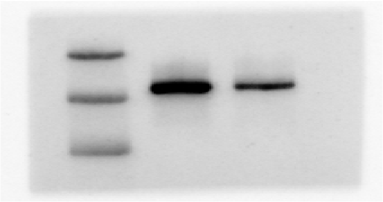

PRDX4

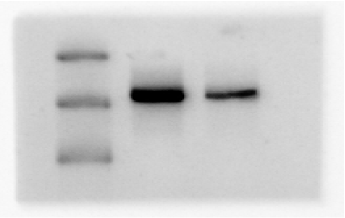

PRDX4

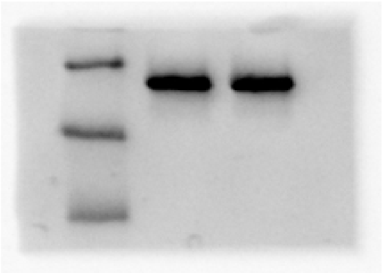

GAPDH

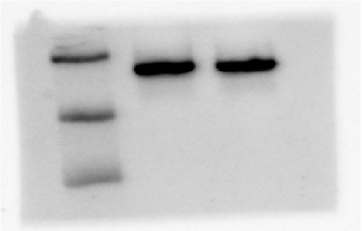

GAPDH

The original western blots of Fig5

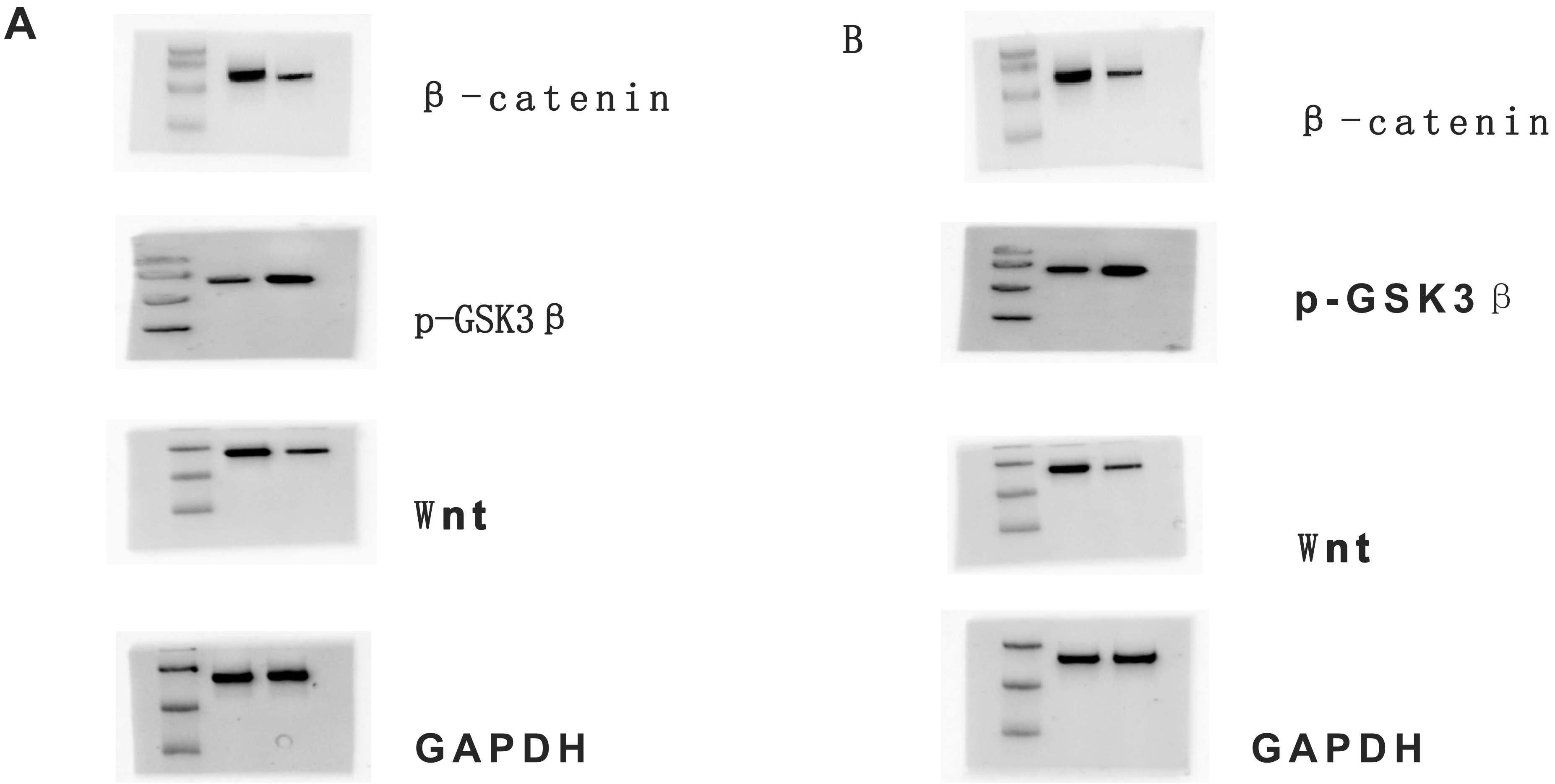

The original western blots of Fig7

A

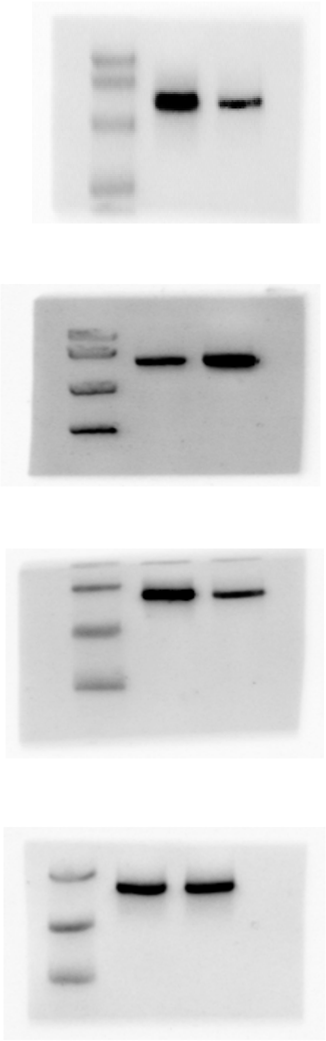

β-catenin

p-GSK3β

Wnt

GAPDH

B

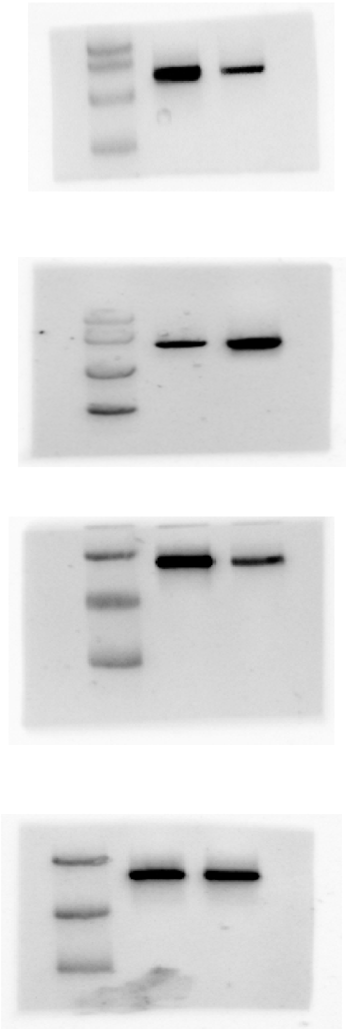

β-catenin

p-GSK3β

Wnt

GAPDH

The original western blots of Fig8

A

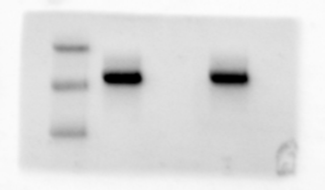

PRDX4

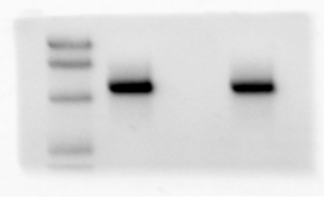

WIF1

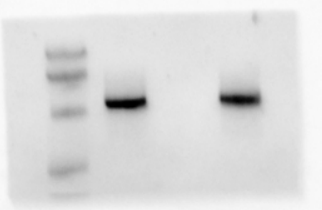

WIF1

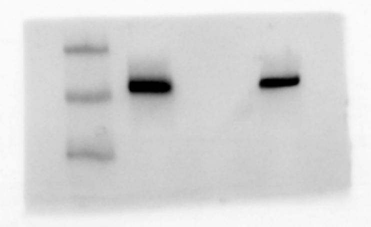

PRDX4

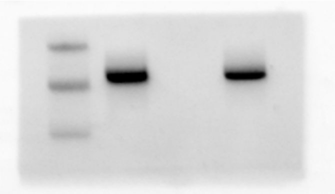

PRDX4

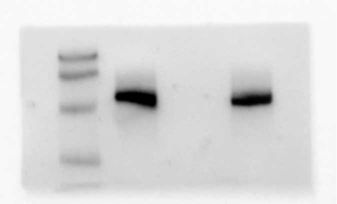

WIF1

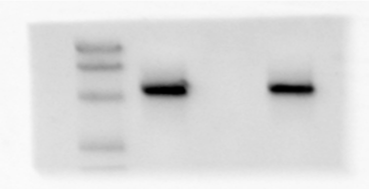

WIF1

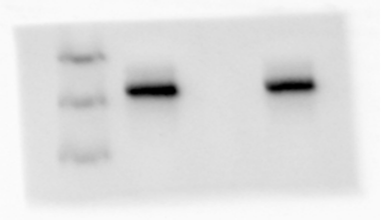

PRDX4

The original western blots of Fig8

C

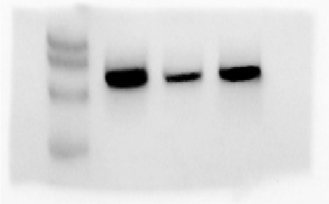

β-catenin

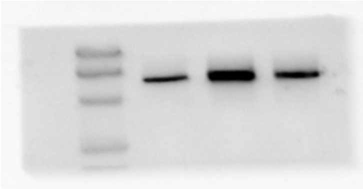

p-GSK3β

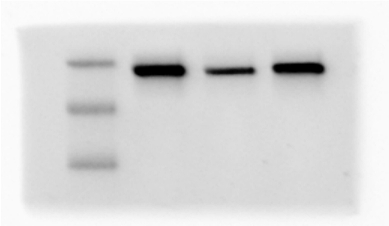

Wnt

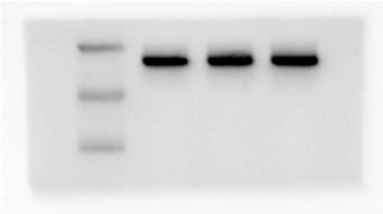

GAPDH

D

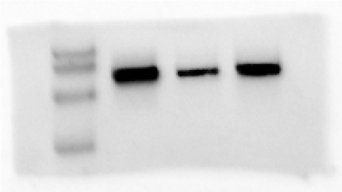

β-catenin

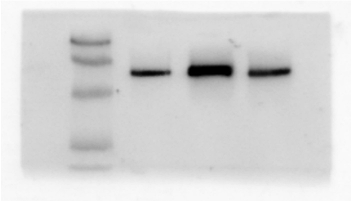

p-GSK3β

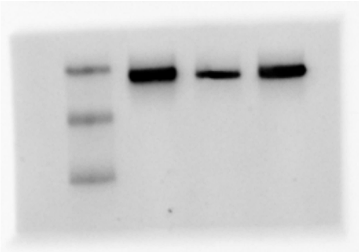

Wnt

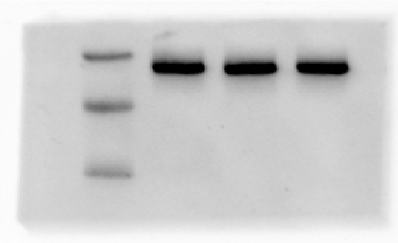

GAPDH

The original western blots of Fig9

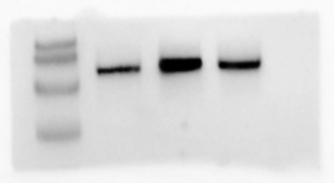

β-cateni
n

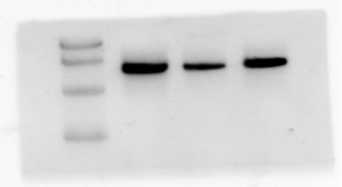

p-GSK3β

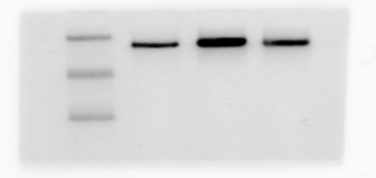

Wnt

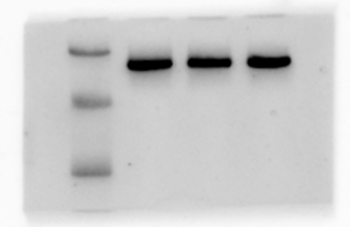

GAPDH

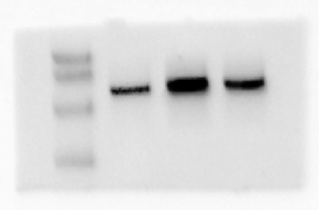

β-catenin

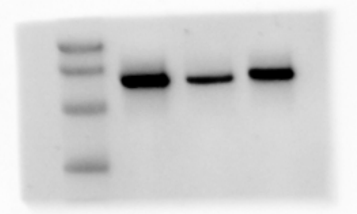

p-GSK3β

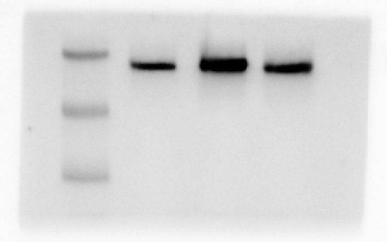

Wnt

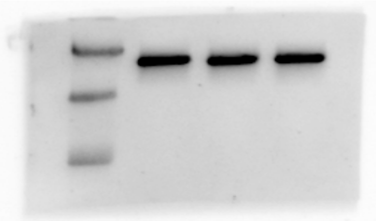

GAPDH
